# Early shape divergence of developmental trajectories in the jaw of galeomorph sharks

**DOI:** 10.1186/s12983-022-00452-1

**Published:** 2022-02-05

**Authors:** Faviel A. López-Romero, Fidji Berio, Daniel Abed-Navandi, Jürgen Kriwet

**Affiliations:** 1 Department of Palaeontology, University of Vienna, Althanstraße 14, Geocenter, 1090 Vienna, Austria; 2 ISEM, CNRS, IRD, EPHE, Univ. Montpellier, Montpellier, France; 3 University of Lyon, Ecole Normale Supérieure de Lyon, Centre National de la Recherche Scientifique, Institut de Génomique Fonctionnelle de Lyon, UMR5242, 46 Allée d’Italie, Lyon, France; 4 Haus des Meeres - Aqua Terra Zoo, Fritz Gruenbaumpl. 1, 1060 Wien, Austria

**Keywords:** Jaw development, Geometric morphometrics, Sharks, Phenotypic trajectory, Elasmobranchs, Catshark, Bamboo shark

## Abstract

**Background:**

The onset of morphological differences between related groups can be tracked at early stages during embryological development. This is expressed in functional traits that start with minor variations, but eventually diverge to defined specific morphologies. Several processes during this period, like proliferation, remodelling, and apoptosis for instance, can account for the variability observed between related groups. Morphological divergence through development is often associated with the hourglass model, in which early stages display higher variability and reach a conserved point with reduced variability from which divergence occurs again to the final phenotype.

**Results:**

Here we explored the patterns of developmental shape changes in the lower jaw of two shark species, the bamboo shark (*Chiloscyllium punctatum*) and the catshark (*Scyliorhinus canicula*). These two species present marked differences in their foraging behaviour, which is reflected in their adult jaw morphology. By tracing the developmental sequence of the cartilage condensation, we identified the onset of cartilage for both species at around stage 31. Other structures that developed later without a noticeable anlage were the labial cartilages, which appear at around stage 33. We observed that the lower jaw displays striking differences in shape from the earliest moments, without any overlap in shape through the compared stages.

**Conclusions:**

The differences observed are also reflected in the functional variation in feeding mechanism between both species. Likewise, the trajectory analysis shows that the main differences are in the magnitude of the shape change through time. Both species follow a unique trajectory, which is explained by the timing between stages.

## Introduction

From an evolutionary perspective, changes in morphology through development are of utmost importance in shaping diversity, either by allowing variation to be displayed or by constraining the possible developmental paths [1–4]. Evidence from molecular and morphological data simultaneously support and question the assumption of common points in development of all vertebrates at which the variation is conserved (i.e., phylotypic stage) [5–9]. Undoubtedly, specific changes at various levels during development can influence and determine the specific morphology and variation [9–12]. Nevertheless, the variation is also constrained within limits that might be imposed to counteract other deleterious effects [9, 11].

Phenotypic divergence in ontogeny between species is a highly dynamic process throughout their comparable stages. In terms of shape changes over time, it is possible to observe distinct patterns: (i) similar trajectories with equal magnitude, (ii) overlapping trajectories differing in magnitude, (iii) parallel trajectories with equal magnitude, or (iv) different starting points that converge over time [13, 14]. The differences in the trajectories can be used to establish whether common moments of convergence exist during ontogeny, and if eventual divergences in shape arise from a common point. For instance, the external morphology of embryos across different amniote classes displays a reduced period of constrained variation from which divergence towards the specific morphologies of the different groups arises [9]. Some of the changes also affect the internal morphology, as developmental mechanisms are acting to determine the final phenotypic outcome. Since some embryonic developmental traits might be regarded as recent adaptive modifications, the selection of traits to be compared among species should consider such biases [15]. An ubiquitous shared trait among jawed vertebrates is the Meckel’s cartilage, whose origin has been suggested either as modification of the first pharyngeal arch of jawless fish, or, conversely, to represent a novelty [16–22]. The developmental function, and eventual fate of the Meckel’s cartilage among vertebrates varies, with some groups retaining it as a functional unit (lower jaw), while in others it represents a transient structure later replaced by dermal bone [23].

**Fig. 1:**
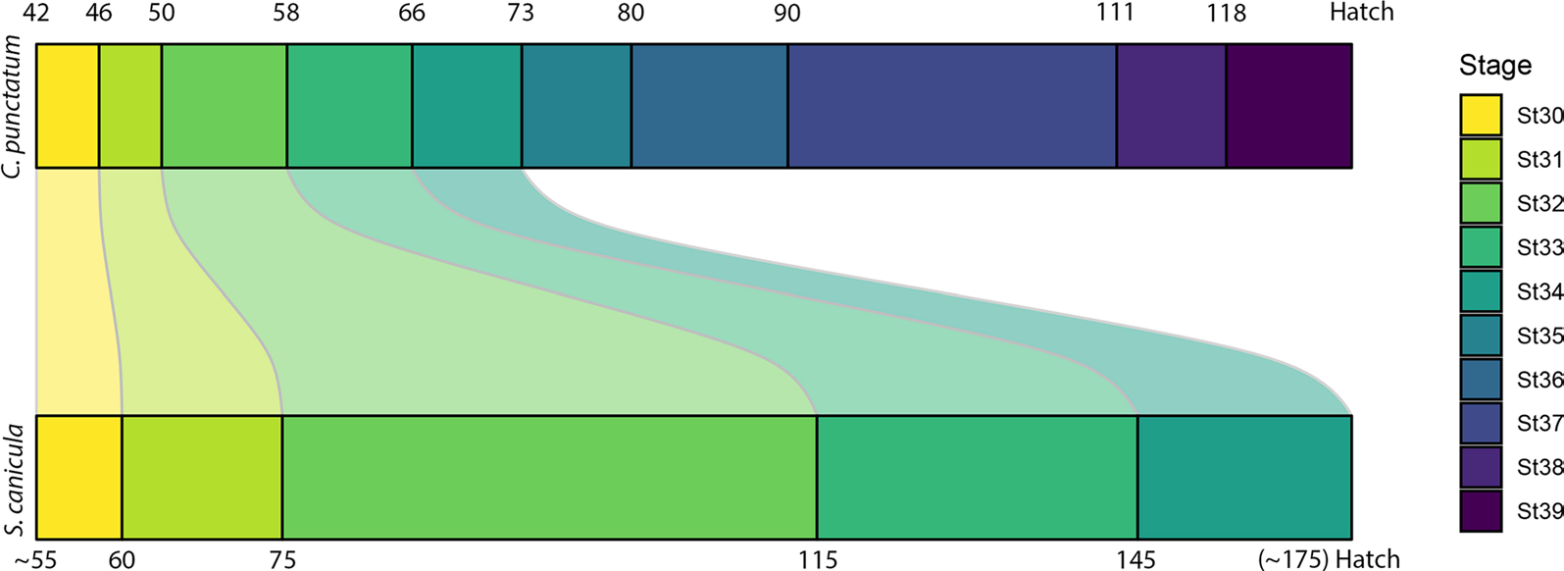
Comparison of embryonic stage duration between the bamboo shark (*Chiloscyllium punctatum*) and the catshark (*Scyliorhinus canicula*). Numbers above and below indicate approximate days after deposition at the start of each stage

Among elasmobranchs, nearly 300 species have been described within the Galeomorph sharks [24, 25], comprising four orders (Heterodontiformes, Orectolobiformes, Lamniformes, and Carcharhiniformes). Within the Orectolobiformes (carpet sharks), the family Hemiscylliidae has two genera: *Hemiscyllium* and *Chiloscyllium*. This family is sister to the nurse sharks (Ginglymostomatidae) and whale shark (Rhincodontidae) [23, 26]. Several families among Orectolobiformes display a particular feeding mechanism of suction feeding, as seen in wobbegongs and notably among the bamboo sharks (*Chiloscyllium* spp.) [27–29], but also filter feeding in the whale shark, for instance [30]. On the other hand, Carcharhiniformes (ground sharks) comprise the most speciose group of galeomorph sharks [25]. Among Carcharhiniformes, the catsharks represent a group with still unresolved phylogenetic relationships [31, 32], however molecular and morphological phylogenetic analyses indicate the family Scyliorhinidae is possibly the basal member within Carcharhiniformes [33]. The family Scyliorhinidae is one of the most speciose groups within Carcharhiniformes with nearly 160 species [24, 25, 34]. In particular, *Scyliorhinus canicula* (hereafter referred to as catshark), is considered a model species in experimental biology [35]. Recently, the orectolobiform bamboo shark has been used in many developmental [36–38], and biomechanical studies [39, 40]. Both species have similar trophic ecologies, although with noticeable differences in their behaviours [35, 41]. Some of these adaptations are expressed in the jaw suspension, which highlights the specialisation of the bamboo shark as suction feeder [41, 42], while the catshark performs mostly grasping assisted with suction prey capture (Pers. Obs. F.B.). Furthermore, the shape difference of the mandibular apparatus between both species is evident in their adult forms [42, 43]. A major difference is also expressed in their early development with the bamboo shark developing at a faster rate than the catshark [37, 44]. Additional developmental stages were described for the bamboo shark, while the later stages in catshark usually last longer, resulting ultimately in the bamboo shark hatching several weeks earlier than the catshark (Fig. 1). However, all comparisons have been based on external features only up to date, which might hinder tracing the development of other skeletal features.

The goal of the present study was to quantify the changes in the lower jaw shape in the bamboo shark and catshark throughout their embryological development to identify similarities and dissimilarities in the timing of trait development. To achieve this, we present here descriptions of the overall developmental sequence of the cartilage formation from the moment of lower jaw condensation at stage 31, until stage 34. We further analysed the shape changes and differences in shape developmental sequences with geometric morphometrics, and with a phenotypic trajectory analysis, respectively. The assessment of shape differences of the different developmental stages highlights the specific morphology of both sharks originating before the cartilage condensation.

## Materials and methods

### Shark embryos

Bamboo shark embryos were obtained from the “Haus des Meeres—Aqua Terra Zoo” in Vienna (Austria). Catshark embryos of different developmental stages were kindly provided by the “Ozeaneum Aquarium” in Stralsund (Germany), the “Musée Océanographique de Monaco” in Monaco (principauté de Monaco), and the University of Montpellier (France). The embryos were fixed in 4% formalin (bamboo sharks) or 4% paraformaldehyde in phosphate buffered saline (PBS) (catsharks) overnight. The fixation was rinsed, and the bamboo shark embryos were transferred through an Ethanol/100 mM Tris pH 7.5 series (25%, 50% and 80%, with 25 mM MgCl_2_), while the catsharks were transferred directly to Ethanol 100%. To estimate the developmental stage, the external morphology was compared with available defined stage tables for each species [33, 40].

### Alcian blue staining

The fixed embryos subsequently were processed in acid-free alcian blue staining following Walker and Kimmel [45] with slight modifications. Prior to staining, the bamboo shark embryos were treated with acetone (100%) to degrease the tissue, while a couple of catsharks at stage 31 were stained with alcian blue/acetic acid/ethanol (Ethanol 80%/ Acetic acid 20%) prior to degrease treatment with trypsin. After staining, the bamboo shark embryos were rehydrated through an Ethanol series up to 25%/100 mM Tris pH 7.5, and for the catsharks in a series of Ethanol 25%/PBS. Afterwards, the specimens were bleached in H_2_O_2_ 3%/KOH 0.5% to remove pigments. Finally, muscles were macerated in 0.5% trypsin/35% sodium borate. The processed embryos were stored in 75% glycerol/0.1% KOH for further studies. All specimens were photographed in ventral view with a Zeiss Discovery V20 stereomicroscope equipped with a Zeiss AxioCam 506 digital camera.

### Landmark and shape analysis

**Fig. 2:**
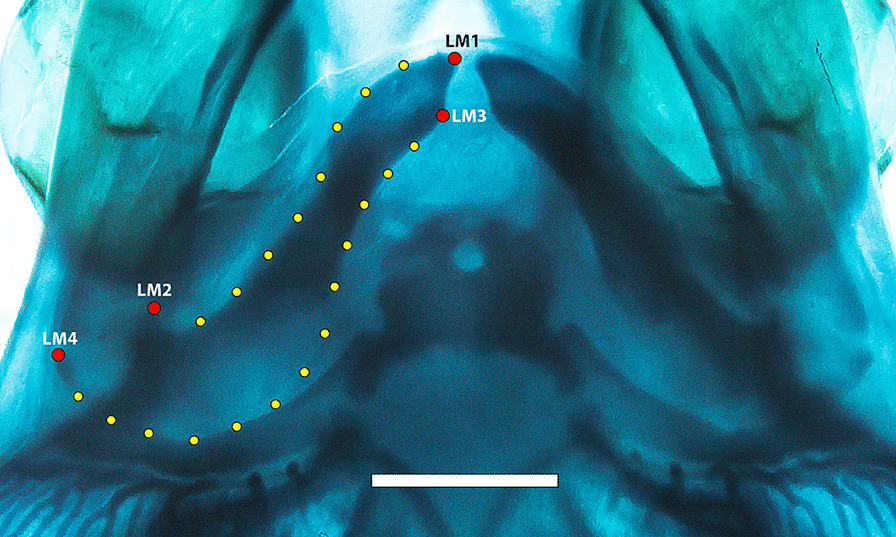
Landmark and semilandmarks coordinates captured on the jaw of *Scyliorhinus canicula* (Stage 33). Red dots represent landmarks, yellow dots represent the semi landmarks to capture the curve shape of the lower jaw. Scale bar = 2 mm

We analyzed a total of 27 embryos of both species, 11 embryos of *C. punctatum* (Stage 31: 1; Stage 32: 3; Stage 33: 3; Stage 34: 4) and 16 embryos of *S. canicula* (Stage 31: 5; Stage 32: 4; Stage 33: 5; Stage 34: 2), ranging from stages 31 to 34. A configuration of landmark coordinates was defined to capture the overall shape of the right Meckel’s cartilage resulting in four landmarks and 21 semilandmarks (Fig. 2), which were digitised using the software tpsDIG2 v.2.18 [46]. The coordinates were then subjected to a generalised Procrustes analysis (GPA) to remove differences in size, position, and orientation [47]. The GPA was performed allowing the semilandmarks to slide to minimise the bending energy [48]. The aligned coordinates were then used for a principal component analysis (PCA) to assess the shape variation for all specimens. The shape variations from the PCA were interpreted by using thin-plate spline deformation grids, which allow observing the shape changes from the mean along the main axes of variation. To determine the number of PCs to be analysed, we used the function getMeaningfulPCs implemented in the Morpho R package [49]. With this method only the first two PCs (accounting for 83.2% of the variance) were deemed meaningful. Another PCA was performed with the shape variables and considering the centroid size to construct a size-shape space [13]. To assess the differences in the mean shapes, while accounting for the size as a covariate and the developmental stage and their taxonomic assignment, we implemented an ANOVA using residuals permutation (999 permutations). Additionally, a phenotypic trajectory analysis was performed on the shape coordinates to estimate changes between species in the path distances, their orientation (measured as angles differences) and the shape changes as the differences in Procrustes distances [50]. The trajectory points were defined by the developmental stages, the earliest ones (stages 31 and 32) were pooled together because of the reduced number of embryos available for those stages, while the remaining (stages 33 and 34) were considered separately. All analyses were performed in the R package geomorph (ver. 4.0.0) [51].

## Results

### Development of the mandibular apparatus

**Fig. 3:**
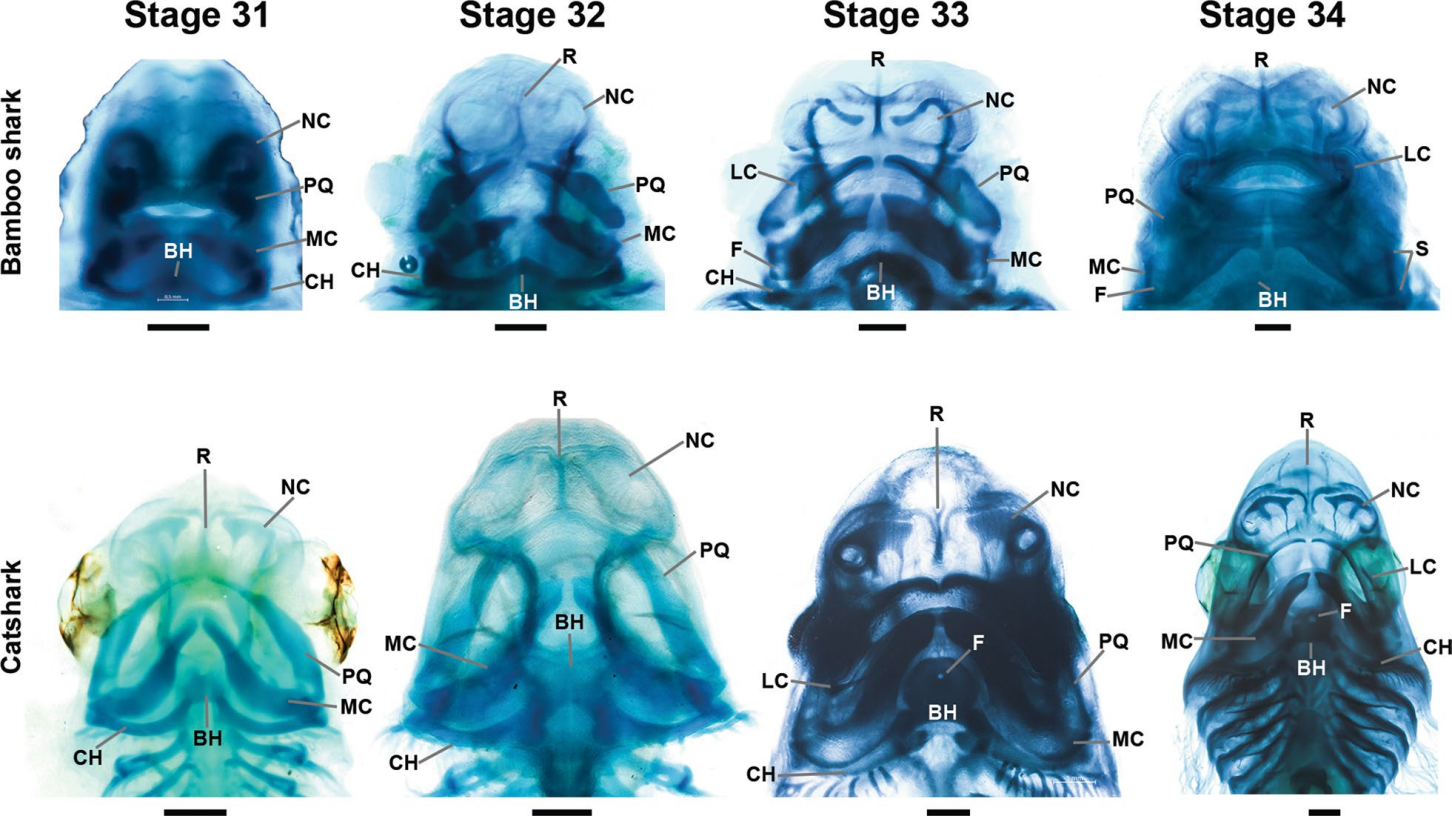
Skeletal development in bamboo shark (*Chiloscyllium punctatum*) and catshark (*Scyliorhinus canicula*) in ventral view. Starting from Stage 31 until stage 34, cartilage stained with alcian blue. *BH* Basihyal, *CH* Ceratohyal, *F* Fenestra, *LC* Labial cartilage, *MC* Meckel’s Cartilage, *NC* Nasal capsule, *PQ* Palatoquadrate, *R* Rostrum, *S* Sustentaculum, *F* Fenestrae. Scale bars = 1 mm

The earliest stage at which any cartilage condensation was possible to detect by alcian blue staining was stage 31 (Fig. 3). All the descriptions are in ventral view.

#### Stage 31

##### Bamboo shark

At this stage, the overall shape of the oral opening is beyond the diamond shape, which occurs at around stage 27 in both species [37, 44], with the rostrum protruding and the mouth commissures pointing backwards. The Meckel’s cartilage in the bamboo shark is slender and very acute at the symphysis, while the palatoquadrate is barely starting to condense (Fig. 3). The ceratohyal is formed and articulates medially to the basihyal.

##### Catshark

In the catshark, the Meckel’s cartilage already is more developed and is “S”-shaped in ventral view, with a broader posterior portion, while the symphysis is slender. The palatoquadrate is already formed and the joints to the Meckel’s cartilage are already present. The ceratohyal also is present and the basihyal has a circular shape.

#### Stage 32

##### Bamboo shark

The Meckel’s cartilage of the bamboo shark becomes more rectangular in shape (Fig. 3), with the anterior portion of the symphysis displaying a sharp end. The palatoquadrate is more evident with a slender symphysis, and the articulation with the lower jaw becomes more prominent. Additionally, a slight condensation of the rostrum is noticeable. The ceratohyal at this stage is more condensed and it becomes more straightened, while the basihyal starts to take a triangular shape.

##### Catshark

The lower jaws are protruding more anteriorly in the catshark, and the symphysis becomes broader antero-posteriorly. Likewise, the palatoquadrate protrudes anteriorly and the rostral cartilage is present. The basihyal at this stage becomes broader compared to the previous stage.

#### Stage 33

##### Bamboo shark

At this stage, the lower jaw becomes more rectangular in shape with a broader symphysis, the posterior part also becomes broader and appears to bend outward and two fenestrae are noticeable in this region, which later are completely closed in adults. The left and right palatoquadrates get closer together medially. Some condensations near the mouth opening are observable at this stage that ultimately will form the labial cartilages. The rostral bar also is now more evident.

##### Catshark

During this stage, the catshark displays a broadening of the posterior part of the Meckel’s cartilage, also the joints to the palatoquadrate become more firmly attached and the symphyseal gap is narrower medially. In the palatoquadrates the symphyseal gap is narrower as well, and strikingly some teeth start to develop both in the palatoquadrate and Meckel’s cartilage. The labial cartilages start to develop as well. The rostrum is now more protruded. The ceratohyal starts to take its position posterior to the Meckel’s cartilage and the basihyal has now a disc shape with a fenestra that is slightly positioned anteriorly.

#### Stage 34

This is the last stage at which we compared both species (Fig. 3), since it is nearly at the hatching time for the catshark, while the bamboo shark still goes through stage 39 in a shorter time span. It is interesting to note that the developmental stages, which are defined by the appearance of specific morphological traits (e.g., yolk reduction, pigment appearance, reduction of median fin folds, gills internalization), are lasting longer in the catsharks than in the bamboo sharks [37].

##### Bamboo shark

At this stage, the symphysis is almost closed (both antimeres meet along the symphysis) in the bamboo shark and the Meckel’s cartilage is now even more rectangular in shape. The posterior part is also now showing sharp angles and the fenestrae perforating the wing-like posterior flanges are still present, making a marked groove forming the sustentaculum. The articulations to the palatoquadrate are now firmly established, and development of teeth also is evident. All three sections (dorsal, medial, and ventral) of the labial cartilages are completely developed.

##### Catshark

In the catshark, the jaws appear now more elongated, with the palatoquadrate extending further to its final dorsal position. Both antimeres of Meckel’s cartilages and palatoquadrates meet medially. The labial cartilages are also evident and finally the basihyal keeps its rounded shape up to this stage.

#### Shape variation of the developing lower jaws

**Fig. 4:**
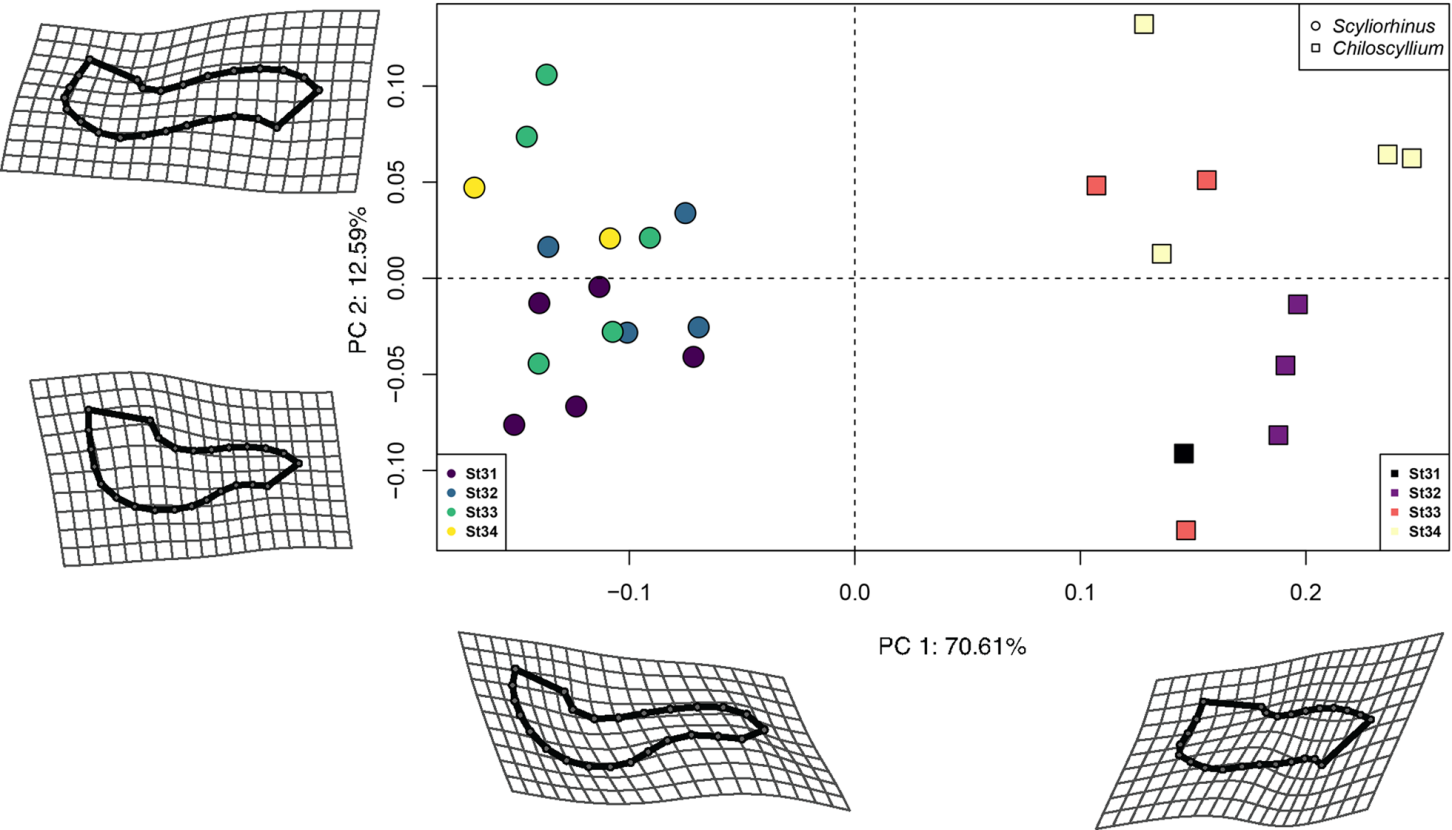
Jaw shape variation through development. Shape-coding indicates the genus and colour gradient their corresponding developmental stage. Deformation grids are along the maximum and minimum values of PC1 and PC2

**Fig. 5:**
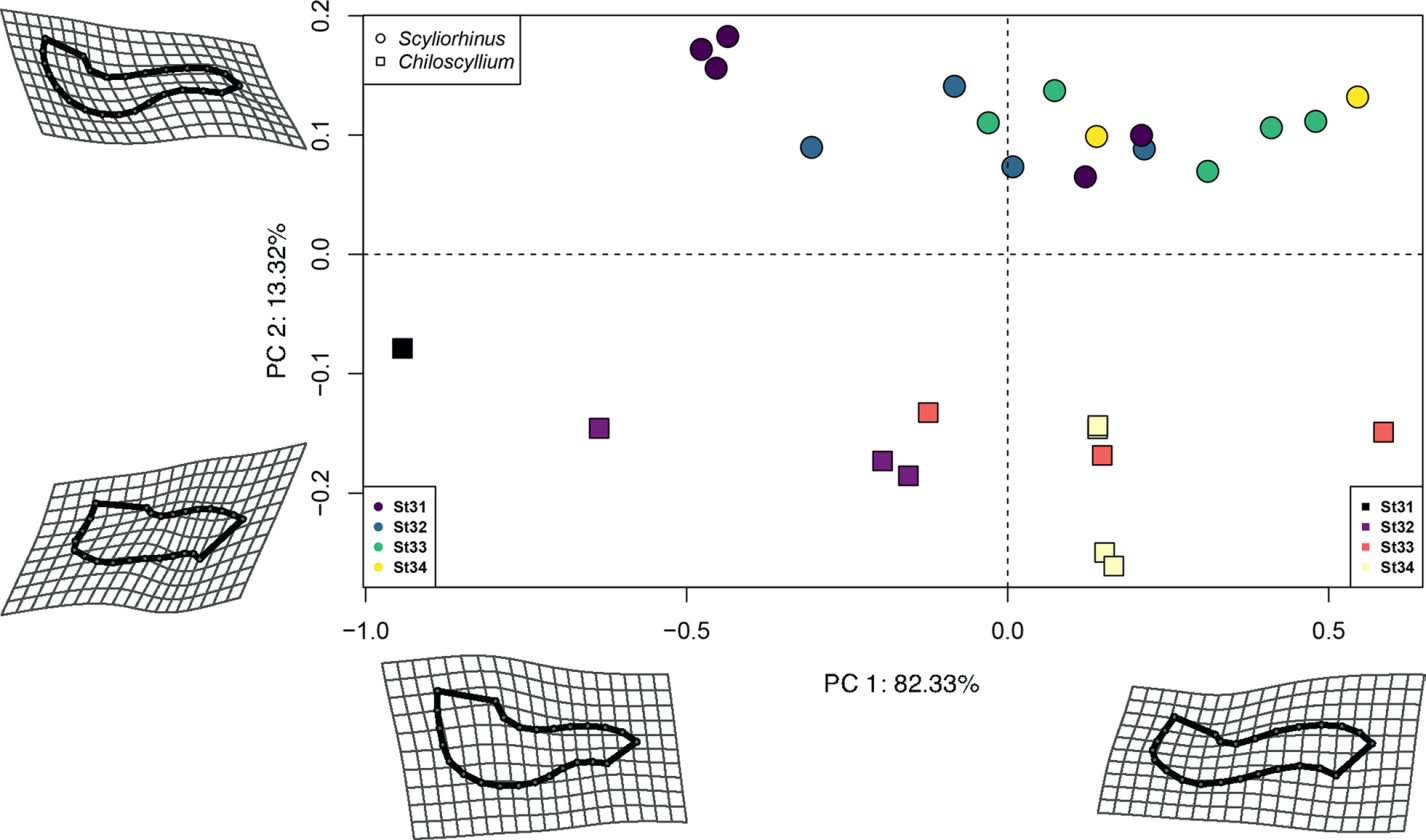
Principal component analysis considering the centroid size regression. Larger embryos are separated along the PC1 at the maximum values, while at PC2 the separation is mainly related by differences from each genus. Shape-coding indicates the genus and colour gradient their corresponding developmental stage

The PCA remarkably shows both species completely separated in the morphospace occupations during each of the developmental stages analysed (Fig. 4). Along the first principal component PC1 (70.61% of the variance) the separation of both species is easily noticeable. In the positive scores, all bamboo sharks are grouped, while the catshark specimens are arranged along the negative scores. Along PC2 (12.59% of the variance) the pattern is less clear, in the negative scores the extreme shapes of the bamboo sharks at early stages are found, while most later stages specimens of the catsharks are in the positive scores. The main differences in shape along PC1 and PC2 are depicted by the deformation grids, which illustrate the marked rectangular shape of the Meckel’s cartilage and deep symphysis in the bamboo shark. In the catshark, the shape changes illustrate that the jaws are more elongated and posteriorly curved in all analyzed stages compared to the bamboo shark. To explore shape changes related to size as the embryos continue developing, the Procrustes coordinates were regressed on the centroid size to obtain a size-shape PCA plot (Fig. 5). Under this scheme, PC1 (82.33% of the variance) shows a more marked separation of the embryos according to their developing stage. In some cases, there are outliers, which correspond to earlier stages plotting ahead of the defined last stage. The PC2 (13.32% of the variance) completely separates both species, with the catshark in the positive scores and the bamboo shark exclusively in the negative scores. The main changes in shape along PC1 for both species are related to the relatively straighter jaws in the negative scores, and subsequently the protrusion of the jaws with a deeper symphysis at the later stages.

**Fig. 6:**
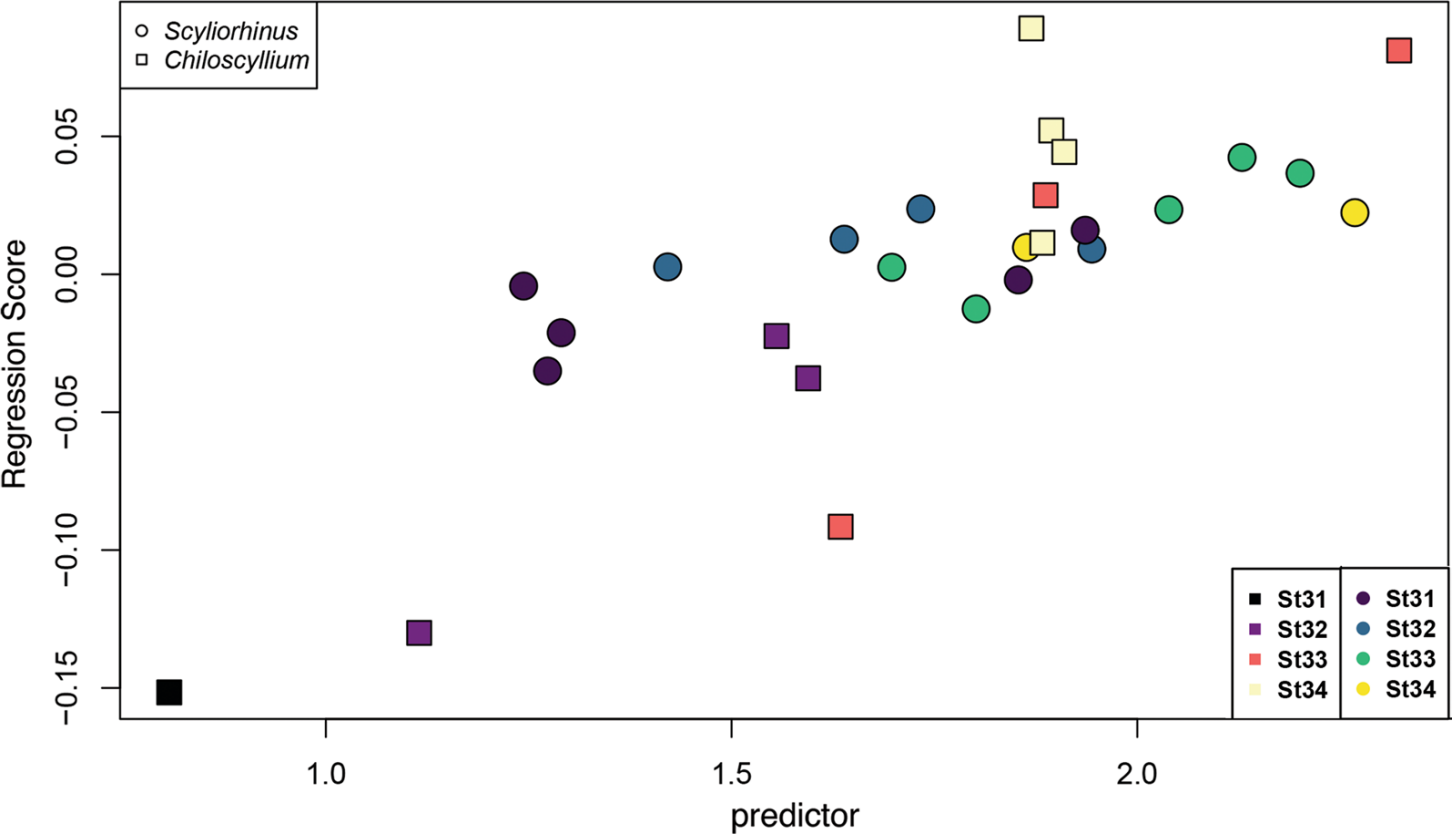
Regression of shape on the logarithm of centroid size (predictor) to visualize shape changes related to the size. Both species follow different slopes through their development. Shape-coding indicates the genus and colour gradient their corresponding developmental stage

**Fig. 7:**
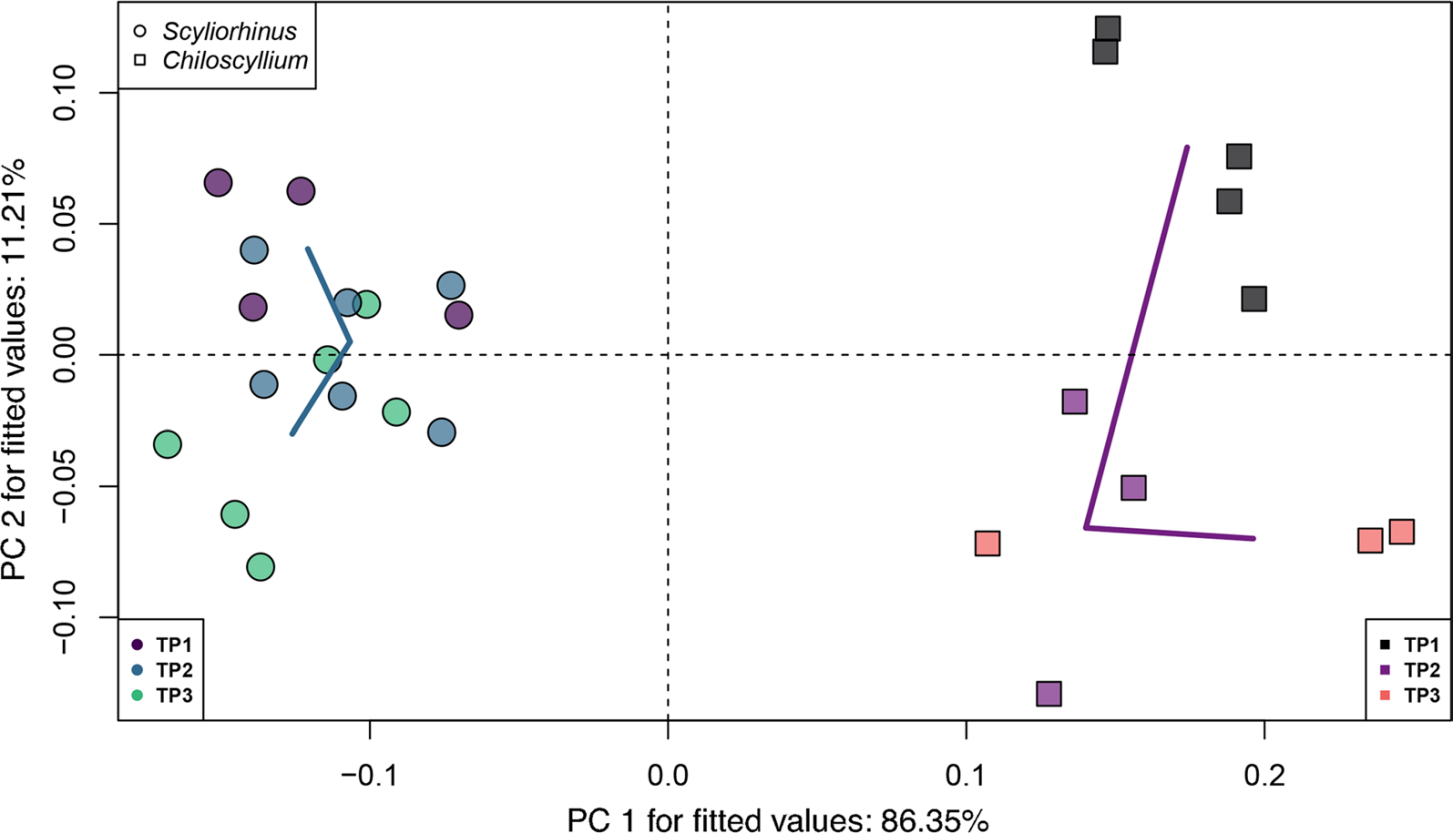
Trajectories analysis plotted on the shape space for both genera, considering the developmental stages. Shape-coding indicates the genus and colour gradient their corresponding developmental stage. Start of the trajectory is indicated by the darker shades. The lighter shade dots indicate the end of the trajectory

To further explore the shape changes during ontogeny between both species, a Procrustes ANOVA was performed with size, genus, and developmental stage as factors. Interestingly, the simplest model of shape and size as covariant is not significant for the shape variation (R^2^ = 0.08224, F = 2.2404, Z = 1.2217, p = 0.122). Likewise, the ANOVA for the interaction of size and stage is not significant (R^2^ = 0.10782, F = 1.1271, Z = 0.35357, p = 0.369). Most of the differences are evident when genus is considered in the model. In this case, most of the variance is explained by genus as a factor, while its interaction with size also is significant (Genus: R^2^ = 0.66541, F = 49.719, Z = 4.0072, p = 0.001; log(Csize)*Genus: R^2^ = 0.03014, F = 2.9352, Z = 2.3831, p = 0.008). A linear regression of the Procrustes coordinates on the logarithm of centroid size shows differences in the shape of the smaller sized bamboo shark, and as the embryos grow, both species follow a unique slope (r = 0.3864, p = 0.014) (Fig. 6). Finally, the shape variation between both species was assessed by a phenotypic trajectories analysis to compare the differences in path distance, angles, and correlation of shape differences. The analysis shows that the main differences are in the magnitude distance between trajectories (Z = 2.25517, p = 0.007). As evident by the PCA of the trajectories analysis (Fig. 7), both species present differences from the earliest stages in development, with no overlap during any analyzed developmental stage.

## Discussion

The ontogenetic shape change comparison between species can occur at various points at comparable stages, with outcomes that suggest shared or divergent trajectories [13, 14, 50]. Overall, our results show a pattern of completely different starting shapes, followed by differences in magnitude of shape change in the trajectories as the development progresses. This suggests that underlying differences between both species are strongly established probably from very early in their development. By observing the embryonic development among elasmobranchs, the external morphology is similar up to specific stages, when divergent features (e.g., pectoral fin expansion in batoids) become more noticeable [37, 44, 52–55]. Differences in developmental timing and trajectories contribute to establish the marked morphological features of the lower jaw in the bamboo shark and catshark. A clear difference among both species is the time span between developmental stages until hatching. Development proceeds in the bamboo shark faster than in the catshark, however, from stage 9 in both species the duration between stages is not so different (ca. 1–2 days), until stage 25 in the catshark when the duration between stages becomes longer compared to the bamboo shark [37]. Meanwhile, the time elapsed between stages 33 and 34 in the catshark for instance, is about four weeks, whereas it is about 8 days in the bamboo shark [44]. Further comparisons beyond stage 34 might be difficult, since in the bamboo shark additional stages are described which does not match the catshark staging table [37]. Another clear difference between both species is the rearing temperature, the catshark in laboratory conditions is reared at 16ºC, whereas the bamboo shark is kept at 25 °C [37, 44]. Although both species face seasonal temperature changes during development in nature, it has been shown that increased rearing temperature directly affects growth rate and survival [56, 57]. The variability in the developmental time among elasmobranchs is noteworthy as some of the species, like the frilled shark, are estimated to have gestation periods between one up to 3.5 years [58].

The developmental sequence of the mandibular arch in the bamboo shark and catshark highlights adaptive differences for prey capture that already are fixed early during development. The jaws, however, not only reflect adaptations in the feeding apparatus, but also other properties correlated to their body plan as in batoids [53]. In other galeomorph groups for instance, different regions of the lower jaw display differences in the extent of mineralization and physical properties related to crushing hard prey during ontogeny [59–61]. Regarding the diet, the bamboo shark species we studied (*Chiloscyllium punctatum*) is considered a generalist with a wide prey spectrum, ranging from fish to crustaceans, and annelids [62]. Prey capture is accomplished by several adaptations in their jaw musculature, and even special modifications in the dentition, as seen in *C. plagiosum* [42]. A noticeable trait difference between *C. punctatum* and *S. canicula* is the sharp bending of the posterior margin of the lower jaw, which forms the sustentaculum in bamboo shark species, but which is not so prominent in the catshark [42, 43]. This is probably a consequence of the specialisation for suction feeding, which is characteristic for Orectolobiformes [27, 28]. Nevertheless, suction feeding also was reported in some catshark species, such as *S. retifer* [63]. To date, the feeding biomechanics of only a single other scyliorhinid, *Cephaloscyllium ventriosum*, has been studied, which was described as a ram feeder [64]. *S. canicula* is often considered a generalist feeder on a wide array of prey items as well [65], although a specific behavior of prey capture has not yet been recorded.

Among amniotes, the craniofacial shape development shows reduced variation early in development, at least in the external morphology, while selecting against morphospace exploration by induced disruptions [9]. In tetrapods like duck, chicken, and quail, the two more closely related (chicken and quail) display a shared static allometry, while the duck shows a divergent pattern of allometry, which accounts for some of the differences among the species [66]. Interestingly, in Smith and collaborators’ study [66], the developmental trajectories between the three bird species reveal that variation at earlier stages is also present before the stages they sampled. The differences in size and shape during development of the Meckel’s cartilage in birds were demonstrated to be under control of the neural crest mesenchyme [67], which impacts the timing of developmental events. Another important feature that accounts for the differences in size is the proliferation of neural crest cells, in which the time between developmental stages of the duck and quail can account for the differences in the size between the species [68]. Additionally, it was observed that mechanical stress during development underlies the morphology of the mandibular apparatus in birds [69]. Meanwhile, in bony fish, gene expression for cartilage formation and re-modelling of the extracellular matrix are mainly responsible for the changes in jaw shape during the ontogeny of morphotypes of the arctic charr *Salvelinus alpinus* [70, 71]. Additionally, the changes in the development rate of the lower jaw were also previously associated with innovations in belonoid fishes [72]. The modulation of Wnt signaling also plays an important role in the development of specialised morphologies in the jaws and craniofacial features in cichlids, while following a conserved ontogenetic trajectory [73, 74]. Other factors like the frequency at which buccal pumping occurs in bony fish is a determinant of the jaw morphology as well [75]. Several studies have documented the onset of morphological specialisations in the skeleton of elasmobranchs during embryological development (e.g., [76–80]). Specifically, regarding cartilage development, the mesenchymal condensations are differentiated into cartilage at stage 31 in catshark [81]. At this stage, cartilage is also detected in our study in bamboo shark. This marks a common moment in mandibular development for both species, which also is shared with another elasmobranch, the little skate *Leucoraja erinacea* [76], and holocephalans [82]. Nevertheless, the shape of the mandibular arch already differs even at this common moment of cartilage condensation between the species we studied, as well as in *Leucoraja* and *Callorhinchus*. We hypothesize that the onset of the differences in shape takes place before cartilage condensation, which is not possible to detect with alcian blue staining. Thus, the spatiotemporal changes in lower jaw morphology are the result of heterotopic and heterochronic changes.

## Conclusions

Our results highlight the importance of the timing in developmental processes among elasmobranchs, and the divergence in shape, which occur even before cartilage differentiation, as a contribution to mandibular arch shape diversity. A period of early shape convergence was not detected by comparing the cartilage development between the species we studied. This pattern can also be seen in other groups such as birds, where despite similarities in trajectories, variation at earlier stages has an evolutionary significance. Among elasmobranchs, investigating other aspects during development can help in understanding trait evolution, particularly in relation to the origin of their body plans.

## Data Availability

Raw landmark coordinates, classifiers variables, and code are available from the corresponding author on reasonable request.
